# Fluorine-Modified Rutaecarpine Exerts Cyclooxygenase-2 Inhibition and Anti-inflammatory Effects in Lungs

**DOI:** 10.3389/fphar.2019.00091

**Published:** 2019-02-07

**Authors:** Chiming Lee, Jiahnhaur Liao, Seuhwa Chen, Chiaohan Yen, Yuchieh Lee, Shihhao Huang, Shengtung Huang, Chunmao Lin, Vincent Hungshu Chang

**Affiliations:** ^1^Graduate Institute of Medical Sciences, College of Medicine, Taipei Medical University, Taipei, Taiwan; ^2^Institute of Biological Chemistry, Academia Sinica, Taipei, Taiwan; ^3^Department of Anatomy and Cell Biology, School of Medicine, College of Medicine, Taipei Medical University, Taipei, Taiwan; ^4^Department of Obstetrics and Gynecology, Taipei Medical University Hospital, Taipei, Taiwan; ^5^Department of Food Technology and Marketing Management, Taipei University of Marine Technology, Taipei, Taiwan; ^6^Graduate Institute of Biochemical and Biomedical Engineering, College of Engineering, National Taipei University of Technology, Taipei, Taiwan; ^7^Department of Biochemistry and Molecular Cell Biology, School of Medicine, College of Medicine, Taipei Medical University, Taipei, Taiwan; ^8^Department of Physiology, School of Medicine, College of Medicine, Taipei Medical University, Taipei, Taiwan

**Keywords:** inflammation, rutaecarpine, ROS, COX-2, KLF-10

## Abstract

Inflammation is the first step that leads to inflammatory cell migration, cytokine release, and myofibroblast formation. Myofibroblasts can deposit excess amounts of extracellular matrix. Cyclooxygenase (COX) inhibitor exhibits strong anti-inflammatory response; however, this is usually achieved with undesirable side effects. In this study, we demonstrated the effects of the fluorine-modified rutaecarpine (RUT), fluoro-2-methoxyrutaecarpine (F-RUT), in inflammatory damage in the lungs. Based on the results, F-RUT retained anti-inflammatory activity both *in vitro* and *in vivo* in lungs. Compared to the parent compound, F-RUT showed better COX-2 suppression as a COX-2-selective inhibitor with lower cytotoxicity, and enhanced molecular reactivity and biological activity. F-RUT was also observed to reduce reactive oxygen species (ROS) generation and inflammatory infiltrating neutrophils in lipopolysaccharide (LPS)-stimulated zebrafish and ovalbumin (OVA)/alum-challenged KLF-10-knockout mouse lungs, respectively. Furthermore, F-RUT ameliorated the respiratory function in OVA/alum-challenged BALB/c mice by maintaining the thickness of the blood-air barrier in mouse lungs. Overall, these data suggest that F-RUT may function as an effective therapeutic agent for inflammation-induced lung dysfunction, and a better selection for pharmaceutical purposes than conventionally used anti-inflammatory agents.

## Introduction

Pulmonary fibrosis is a complex disease with limited therapeutic options. Inflammation is the critical first step during the progression of fibrosis and this triggers coagulation, and fibroblast proliferation, and activation (Wynn, 2011). These processes lead to the transformation of myofibroblasts from a variety of sources including bone marrow fibrocytes, resident fibroblasts, and epithelial cells, through epithelial-to-mesenchymal transition (EMT). Excess extracellular matrix produced by myofibroblasts causes scar formation, which leads to the disruption of gas exchange and irreversible destruction within the lungs. During inflammation, infiltrating neutrophils and cytokines are released. Usually, this results in irreversible airway remodeling and pulmonary fibrosis. An anti-inflammatory drug that inhibits neutrophil movement may therefore provide insights for the prevention of pulmonary fibrosis (Schilter et al., 2015).

Cyclooxygenase (COX) isozymes are responsible for the generation of prostaglandin (PG) from arachidonic acid, and PG is known to mediate the inflammatory response and sustain homeostatic function. For example, the COX-2 isozyme is induced by inflammatory stimuli, and is an important source of PGs such as prostaglandin E_2_ (PGE_2_), prostaglandin D_2_ (PGD_2_), prostacyclin (PGI_2_), and prostaglandin F_2_α (PGF_2_α), during inflammation. Another member of the COX family, COX-1, is also constitutively expressed in a variety of cell types to perform housekeeping functions such as cellular homeostasis and gastric epithelial cytoprotection (Ricciotti and FitzGerald, 2011). The pro-inflammatory cytokine, interleukin (IL)-1, also mediates the induction of COX-2 expression and the activation of nuclear factor (NF)-κB, extracellular signal-regulated protein kinase (ERK), p38, and protein kinase C (PKC) signaling pathways in intestinal myofibroblasts (Mifflin et al., 2002). In fact, myofibroblast migration is stimulated by tumor necrosis factor (TNF)-α, a pro-inflammatory cytokine, through the COX-2 and heat-shock protein 27 (Hsp27) pathways (Saini et al., 2016; Chu et al., 2017). Since elevated inducible COX-2 is often found at sites of inflammation and malignant transformation, it is considered an ideal target for the anti-inflammatory activity of non-steroidal anti-inflammatory drugs (NSAIDs) (Taketo, 1998). In addition, blockage of COX-1 and COX-2 may result in adverse effects in the gastrointestinal tract (Fiorucci et al., 2003). A new type of NSAID and COX-2 selective inhibitor may serve as the preferred option for inflammation (Warner and Mitchell, 2002). Two commercial anti-fibrotic drugs, nintedanib, and pirfenidone, were found to possess anti-inflammatory activity and COX-2 suppression effect (Choi et al., 2013; Alves et al., 2018). However, side effects of both drugs such as gastrointestinal disturbance, gastritis, diarrhea, and vomiting remain concerning for patients, especially those who require high-dose therapeutic treatment.

Rutaecarpine (RUT) was isolated from the traditional Chinese medicinal herb, *Evodia rutaecarpa*. RUT exhibits biological activities including anti-inflammation, a cardio-tonic effect, and anti-coagulation (Sheu et al., 2000; Kobayashi et al., 2001; Choi et al., 2006). Recent studies have demonstrated that RUT can suppress cell migration and COX-2 activity (Moon et al., 1999; Chiou et al., 2011). In our previous work, we synthesized the RUT derivative, fluoro-2-methoxyrutaecarpine (F-RUT), and demonstrated its anti-inflammatory effect in macrophages (Lee et al., 2017). In this study, we further explored its therapeutic effect in the reduction of inflammatory damage in lungs. In addition, we aim to present a remedy for the side effects of the COX-2-selective inhibitors.

## Materials and Methods

### Chemicals and Reagents

All chemicals were purchased from Sigma-Aldrich (St. Louis, MO, United States) and Thermo Fisher Scientific (North Ryde, Australia) without further purification unless otherwise stated.

### Synthesis of Fluoro-2-Methoxyrutaecarpine (F-RUT)

Fluoro-2-methoxyrutaecarpine (F-RUT) was synthesized as previously reported (Lee et al., 2017). In brief, 2-amino-4,5-dimethoxybenzoic acid (0.24 g, 1.5 mmol) was dissolved in toluene (5 mL, 0°C) and added dropwise to thionyl chloride (0.87 mL, 7.4 mmol). The mixture was heated to 70–80°C and stirred for 1 h. Further heating for reflux was performed for 10 min then cooled to 23°C and concentrated. The resulting residue was restored in toluene (5 mL), and added to 2,3-piperidinedione-3-(4-fluorophenyl) hydrazone (0.1 g, 0.5 mmol). The reaction mixture was heated for reflux and stirred overnight. The solution was then concentrated using a rotary evaporator; 10% sodium carbonate aqueous was added (200 mL), and the reaction was extracted by dichloromethane (3 × 200 mL). The organic layer was dried over anhydrous MgSO_4_. Solids were filtered through a fritted Büchner funnel, and the solution was concentrated under reduced pressure. The residue was purified by column chromatography (EA:hexane = 1:2) to obtain solid F-RUT (Supplementary Scheme 1).

### Cell Culture

Human lung epithelial cells, H460 and CL1-3 cells, were grown in RPMI 1640 medium supplemented with 10% fetal bovine serum (FBS) and antibiotics (100 U/mL penicillin and 100 μg/mL streptomycin) at 37°C in a 5% CO_2_ humidified atmosphere.

### Cell Viability Assay

We used an MTT assay to determine cell viability based on the conversion of the yellow tetrazolium salt to the purple formazan product (Suk et al., 2013). Cells (10^4^ cells/well) were grown in standard culture medium in a 96-well plate and treated with RUT and F-RUT (0–20 μM) for 24 h. MTT stock solution (5 mg of MTT/mL of phosphate-buffered saline; PBS) was added to the growing cultures for 2 h. The absorbance was measured at 560 nm with a spectrophotometer. DMSO alone was measured as a reading control. Data are presented as mean ± standard deviation (SD) of five independent experiments.

### Protein Expression Analysis

Protein expression was determined by a western blot assay. Protein samples were separated and resolved by sodium dodecyl sulfate polyacrylamide gel electrophoresis (SDS-PAGE) and electrotransferred onto a polyvinylidene difluoride (PVDF) membrane. The membrane was incubated with primary antibody at 4°C overnight, followed by a second incubation with a horseradish peroxidase (HRP)-conjugated secondary immunoglobulin G (IgG) antibody; immunoreactive bands were visualized with PerkinElmer (Waltham, MA, United States) enhanced chemiluminescent reagents (Yu et al., 2011).

### Cell Migration Assay

Cell migration was evaluated by individually growing cells in 24-well transwell plates (with 8-μm-pore filters, Merck Millipore, Burlington, MA, United States) (Lin et al., 2010). Cells (2 × 10^4^cells/well) were cultured for 24 h in RPMI 1640 medium with 10% FBS. F-RUT at 20 μM was placed in the upper chamber of the transwell with cells for an additional 24 h; medium containing 10% FBS only was placed in the lower chamber. At the end of incubation, non-migrated cells in the upper chamber were removed with a cotton swab; cells that had migrated to the opposite side of the filter were fixed with 4% formaldehyde and stained with 2% crystal violet. Stained cells were counted and photographed under a phase-contract optical microscope at 200× magnification. Data are presented as mean ± SD of three independent experiments.

### Animal Experiments

Six-week-old Krüppel-like factor 10-knockout (KLF-10-KO) mice were provided by Dr. Vincent Hung-Shu Chang (The Ph.D. Program for Translational Medicine, College of Medical Science and Technology, Taipei Medical University, Taipei, Taiwan). BALB/c mice (6 weeks of age) were obtained from the Animal Center of the College of Medicine, National Taiwan University (Taipei, Taiwan). Mice were sensitized intraperitoneally with 20 μg of ovalbumin (OVA) emulsified in 2 mg of aluminum hydroxide (alum) in 200 μL PBS on day 0, and boosted with 50 μg of OVA emulsified in 4 mg of aluminum hydroxide on days 14 and 28. F-RUT was orally administered on days 30, 32, 34, 36, and 38. For post-challenge, all mice were treated intranasally with OVA (100 μg in a total volume of 40 μL PBS) on days 40, 41, 42, and 43. At 24 h after the last OVA challenge, mice were perfused with 4% paraformaldehyde and sacrificed, and the organs were collected. All experimental procedures were reviewed and approved by the Institutional Animal Care and Use Committee or Panel at Taipei Medical University (LAC-2015-0273). Lung tissues were fixed in 4% paraformaldehyde (sc-281692; Santa Cruz Biotechnology, Dallas, TX, United States) and embedded in paraffin. Tissues were sliced to obtain 5-μm thick sections, and stained with hematoxylin and eosin (H&E) solution to detect inflammation, and Masson trichrome stain to detect collagen formation (Huang et al., 2008). To evaluate collagen formation, the intensity of collagen was normalized by the total area around the bronchi in six random regions from each sample (*n* = 3), and the average percentage was calculated.

### Measurement of Reactive Oxygen Species (ROS) Production in Zebrafish Larvae

Embryos (*n* = 9) 3 days post-fertilization (dpf) were transferred to individual wells of a 24-well plate and maintained in embryo media containing sterile distilled water (vehicle control), 5 μg/mL F-RUT (final concentration) alone, 10 or 20 μg/mL lipopolysaccharide (LPS) (final concentration), or 5 μg/mL F-RUT for 2 h followed by treatment with LPS, except for larvae in the control group. For up to 4 dpf, the generation of reactive oxygen species (ROS) in zebrafish larvae was analyzed using the fluorescent probe dye, 2′,7′-dichlorofluorescin diacetate (DCF-DA). Larvae were transferred to 24-well plates, incubated with a DCF-DA (20 μg/mL) solution for 1 h in the dark at 28.5°C, and then anesthetized using 1-phenoxy-2-propanol (1/500 dilution, Acros Organics, Morris Plains, NJ, United States). Images of stained larvae were observed for ROS generation under a fluorescence microscope, and the fluorescence intensity of individual larvae quantified at an excitation wavelength of 485 nm and an emission wavelength of 535 nm using a spectrophotometer and TissueQuest analytical software (TissueGnostics, Vienna, Austria), respectively. ROS generation was calculated by comparing the fluorescence intensity of treated larvae to that of the controls (Kwon et al., 2017).

### Computational Molecular Docking

Docking experiments were performed using the Discovery Studio 4.5 software package (Accelrys Software, San Diego, CA, United States). Structures of the analyzed compounds were built by ChemBioDraw Ultra 12.0 (PerkinElmer, Waltham, MA, United States) and were optimized by energy minimization before docking. Human COX-2 (hCOX-2; PDB:5IKQ) and ovine COX-1 (oCOX-1; PDB: 5WBE), downloaded from the Protein Data Bank^1^, were used in the docking experiments. Before docking, hydrogen atoms were added to the unoccupied valence of the apo hCOX-2 and apo oCOX-1 structures. The surface of the protein was selected as the docking region. CDOCKER was used in subsequent docking experiments. Structural figures were generated using the programs LigPlot2 (Wallace et al., 1995) and PyMOL (Schr€odinger, New York, NY, United States).

### Cyclooxygenase (COX)-Inhibitory Assay

A cyclooxygenase (COX) activity assay was performed according to Yang et al. (2001). Inhibitory actions of F-RUT and RUT toward COX-1 and COX-2 activities were individually determined using a fluorometric COX inhibitor screening assay kit as recommended by the manufacturer (catalog no. K548-100 for COX-1, and K547-100 for COX-2, Biovision, Milpitas, CA, United States). The assay directly detects fluorometric PGG2 generated by the COX enzyme at Ex/Em = 535/587 nm using a microplate reader (Thermo Scientific, Waltham, MA, United States). The average fluorescence was calculated for all samples (*n* = 3) to determine percent inhibition.

### Transmission Electron Microscopy (TEM)

Mice lungs were harvested and fixed in 2.5% glutaraldehyde and 2% paraformaldehyde in 0.1 M cacodylate buffer (pH 7.0) for 8 h at 25°C. The lungs were postfixed in 1% osmium tetroxide for 1 h and dehydrated in sequential steps using ethanol (75, 80, 90, and 95% twice, and 100% three times), then embedded in a resin (TAAB Lab Equipment, Aldermaston, United Kingdom). Ultrathin 80-nm sections were subsequently cut using a diamond knife on a Leica EM UC7 ultramicrotome. Images were captured using a TEM (Hitachi HT-7700; Tokyo, Japan) at 75 keV. Blood-air barrier thickness, composed of the alveolar epithelium (EP), capillary endothelium (EN), and basal lamina (BL), was measured in six random regions from each sample (*n* = 4 or 5 per group), and average thickness was calculated.

### Statistical Analysis

All data presented in this article were generated from at least three biological replicates to meet the requirements for statistical validity. One-way analysis of variance (ANOVA) followed by Tukey’s test was used to determine significant differences among groups unless otherwise stated. All data are presented as mean ± SD. Differences were considered significant when *p* < 0.05. All statistical analyses were performed using GraphPad Prism 5 software (GraphPad, San Diego, CA, United States).

## Results

### Fluoro-2-Methoxyrutaecarpine (F-RUT) Exhibits Better Inhibitory Effect on COX-2 and Lower Cytotoxicity Than RUT in Lung Epithelial Cells

**FIGURE 1:**
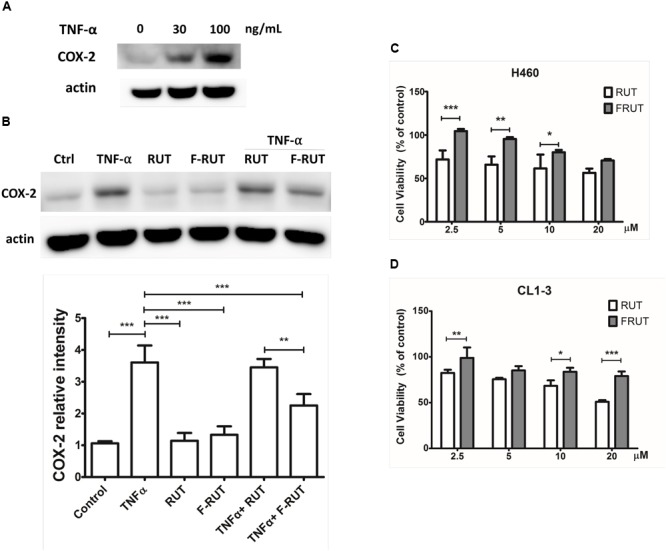
Fluoro-2-methoxyrutaecarpine (F-RUT) exhibited a better suppression effect on tumor necrosis factor (TNF)-α induced cyclooxygenase (COX)-2 and lower cytotoxicity in lung epithelial cells than RUT. **(A)** The protein expression of COX-2 was induced by TNF-α in a concentration-dependent manner. **(B)** Effects of F-RUT and rutaecarpine (RUT) on COX-2 protein expression in TNF-α-treated H460 lung cells by a western blot analysis. Cells were stimulated with 30 ng/mL TNF-α and treated with RUT and F-RUT for 24 h at 5 μM. Cell viability of H460 **(C)** and CL1-3 cells **(D)** was measured following treatment with RUT and F-RUT for 24 h at 2.5–20 μM (by a two-way ANOVA statistical analysis followed by the Bonferroni post-test to determine significant differences among groups. ^∗^*p* < 0.05, ^∗∗^*p* < 0.01, ^∗∗∗^*p* < 0.001; *n* = 3 or 4 per group).

In our previous study, we established the anti-inflammatory function of F-RUT in macrophages (Lee et al., 2017). The stimulation of epithelial cells by inflammatory stimuli plays a critical role during the progression of lung fibrosis. TNF-α, a pro-inflammatory cytokine, is known to stimulate myofibroblast migration via COX-2 (Saini et al., 2016). In addition, it induces the translational expression of COX-2 at 30 and 100 ng/mL in H460 lung epithelial cells (Figure 1A). Five micromoles of F-RUT exhibited a significantly greater reduction effect than 5 μM RUT (Figure 1B; ^∗∗^*p* < 0.01). In contrast, RUT did not influence COX-2 expression following co-treatment with TNF-α (30 ng/mL) (Figure 1B). More interestingly, the cytotoxicity of F-RUT was notably lower than RUT in both H460 (Figure 1C) and CL1-3 lung epithelial cells (Figure 1D) (^∗^*p* < 0.05, ^∗∗^*p* < 0.01, ^∗∗∗^*p* < 0.001, compared to the RUT-treated group). The 50% inhibitory concentration (IC_50_) values are presented in Table 1.

**Table 1:** IC_50_ values of rutaecarpine (RUT) and fluoro-2-methoxyrutaecarpine (F-RUT) in lung epithelial cells.

IC_50_ [μM]^a^	RUT	F-RUT	*p*-value
H460	20.43 ± 5.42	65.53 ± 2.42	0.0002
CL1-3	20.55 ± 1.97	50.95 ± 3.96	0.00028

*^*a*^50% inhibitory concentration (IC_*50*_) values are presented as mean ± standard error of mean calculated from three independent experiments*.

### Fluoro-2-Methoxyrutaecarpine (F-RUT) Suppresses Cell Migration in Lung Epithelial Cells

**FIGURE 2:**
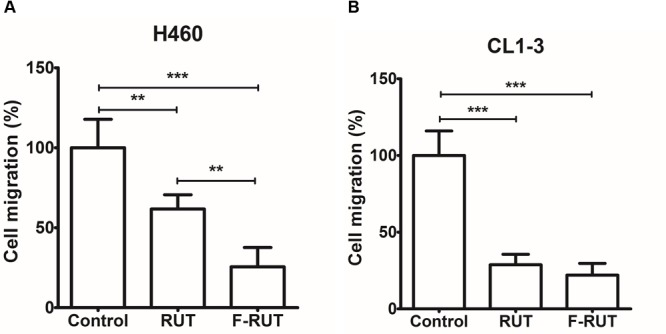
Fluoro-2-methoxyrutaecarpine (F-RUT) suppressed H460 **(A)** and CL1-3 **(B)** lung epithelial cell migration. Cell migration was detected following F-RUT or rutaecarpine (RUT) treatment for 24 h at 20 μM, and migrating cells were counted (^∗∗^*p* < 0.01, ^∗∗∗^*p* < 0.001; *n* = 5 per group).

Cyclooxygenase (COX)-2 is known to trigger myofibroblast migration by TNF-α (Saini et al., 2016). To clarify whether F-RUT can specifically inhibit the function of COX-2, a transwell assay was performed with H460 and CL1-3 lung epithelial cells. A 20 μM concentration of RUT and F-RUT was added to H460 and CL1-3 cells for 24 h to evaluate cell migration (Figure 2A,B, respectively) and the number of migrating cells quantified using an imaging software. Compared to RUT, which produced averages of 61.7 and 28.73% for cell migration effects, F-RUT showed an average of 25.53 and 22.02% in H460 and CL1-3 cells, respectively (Figure 2A,B, respectively). Therefore, F-RUT showed a significant suppressive effect on cell migration compared to RUT (^∗∗^*p* < 0.01, ^∗∗∗^*p* < 0.001 compared to the control group in H460 and CL1-3 cells, respectively).

### Fluoro-2-Methoxyrutaecarpine (F-RUT) Can Effectively Bind to COX-2 as Modeled by Biomolecular Simulation

**FIGURE 3:**
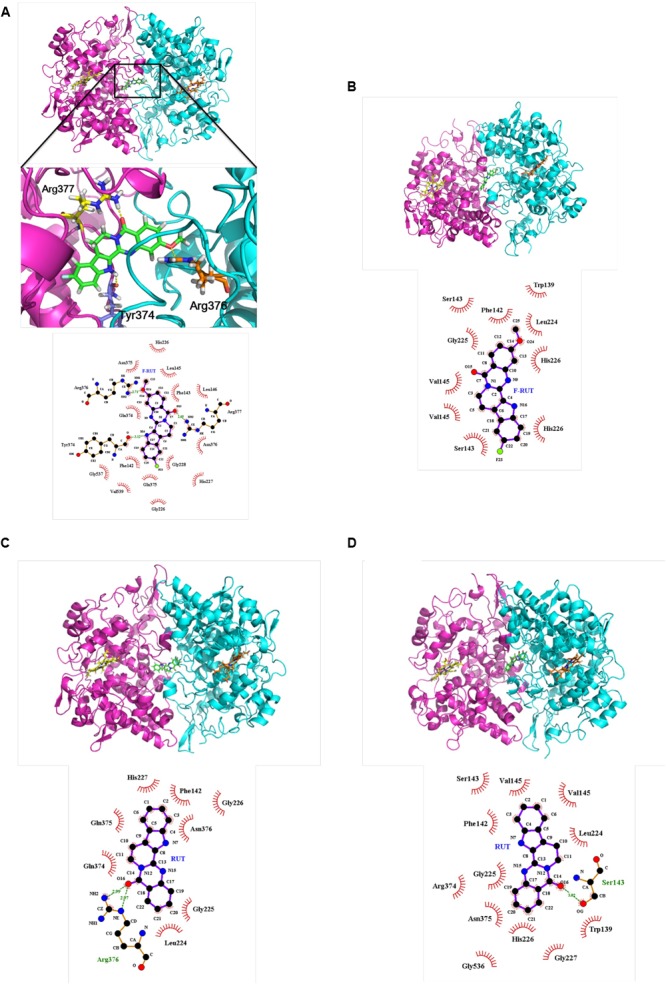
Proposed structural models of human cyclooxygenase (hCOX)-2-fluoro-2-methoxyrutaecarpine (F-RUT) **(A)**, ovine (o)COX-1-F-RUT **(B)**, hCOX-2-rutaecarpine (RUT) **(C)**, and oCOX-1-RUT **(D)** complexes. 3D models of oCOX-1 and hCOX-2 showed the dimeric form of the complexes. The compounds, RUT and F-RUT (green), were bound to the interface of the oCOX-1 and hCOX-2 subunits. The orange and yellow molecules are heme groups. Interactions of RUT and F-RUT with oCOX-1 and hCOX-2 are shown by LigPlot. Carbon, oxygen, and nitrogen atoms are, respectively, shown as black, red, and blue circles; the fluorine atom is shown as light-green. Red eyelashes indicate residues involved in hydrophobic interactions. Hydrogen bonds are represented by the green dash lines.

A molecular docking analysis was performed to predict the strength of bonding forces and identify the best geometrical arrangement between F-RUT and COX-2 (Rudnitskaya et al., 2010). The proposed 3D structural models of the hCOX-2-F-RUT complex showed a dimeric form of the complex. F-RUT (green) was bound at the interface of the hCOX-2 subunits and three amino acid residues of hCOX-2 formed hydrogen bonds with F-RUT (Figure 3A). Arg377 of subunit 1 was observed to bind to N- of the indole moiety, while Arg376 and Tyr374 of subunit 2 was observed to bind to methoxy group of 14C, and O- of the carbonyl group, respectively. A substituted fluoro-group was observed to be surrounded by Gly228, Gln375, and Phe142 (Figure 3A) via hydrophobic interactions. The 3D model of the oCOX-1-F-RUT complex also showed a dimeric form of the complex and a hydrogen bond did not interact with F-RUT (Figure 3B). The calculated binding energy of the hCOX-2-F-RUT complex was -36,573.281 kcal/mol, while that of the oCOX-1-F-RUT complex was -32,686.354 kcal/mol (Table 2). F-RUT was observed to display COX-2-selective inhibition. The lead compound, RUT, demonstrated a similar binding pose (Figure 3C,D) and displayed COX-2-selective inhibition with binding energies of -36,466.510 kcal/mol to hCOX-2 and -32,578.399 kcal/mol to oCOX-1 (Table 2).

**Table 2:** Calculated binding energy of cyclooxygenase (COX)-rutaecarpine (RUT) derivative complexes.

Total energy (kcal/mol)	F-RUT	RUT
oCOX-1	–32,686.354	–32,578.399
hCOX-2	–36,573.281	–36,466.510

*o, ovine; h, human; F-RUR, fluoro-2-methoxyrutaecarpine.*

### Fluoro-2-Methoxyrutaecarpine (F-RUT) Is a COX-2–Selective Inhibitor

**FIGURE 4:**
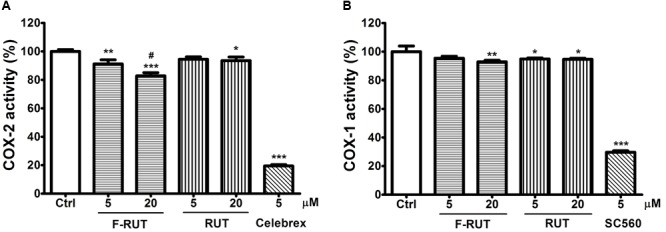
Fluoro-2-methoxyrutaecarpine (F-RUT) suppressed cyclooxygenase (COX)-2 activity. The inhibitory activities of rutaecarpine (RUT) and F-RUT against COX-1 **(A)** and COX-2 **(B)** were measured by the level of prostaglandin generated at 5 and 20 μM. Celebrex and SC560 were inhibitors of COX-2 and COX-1, respectively. (^∗^*p* < 0.05, ^∗∗^*p* < 0.01, ^∗∗∗^*p* < 0.001, compared to control group; ^#^*p* < 0.001, compared to 20 μM RUT group; *n* = 3 per group).

Rutaecarpine (RUT), the lead compound of F-RUT, inhibits COX-2 activity and demonstrates anti-inflammatory capability in bone marrow–derived mast cells (Moon et al., 1999). Chaiamnuay et al. (2006) also reported that traditional NSAIDs can be differentiated based on different inhibition ratios of COX-1 and COX-2. To further evaluate the effect of F-RUT on COX activities, generation of the byproduct, prostaglandin, was measured. At 20 μM, F-RUT produced a 20% reduced effect on COX-2 (Figure 4A; ^∗∗∗^*p* < 0.001, compared to the control group). Compared to RUT and at 20 μM, F-RUT exhibited a stronger inhibitory effect (Figure 4A; ^#^*p* < 0.001). Similar to RUT, F-RUT showed low inhibitory effects against COX-1 (Figure 4B). These data demonstrate the specificity of F-RUT against COX-2 (Supplementary Table S1), and are consistent with the molecular docking results. F-RUT would therefore serve as a more-effective NSAID candidate than RUT.

### Fluoro-2-Methoxyrutaecarpine (F-RUT) Suppresses ROS Generation in LPS-Induced Zebrafish

**FIGURE 5:**
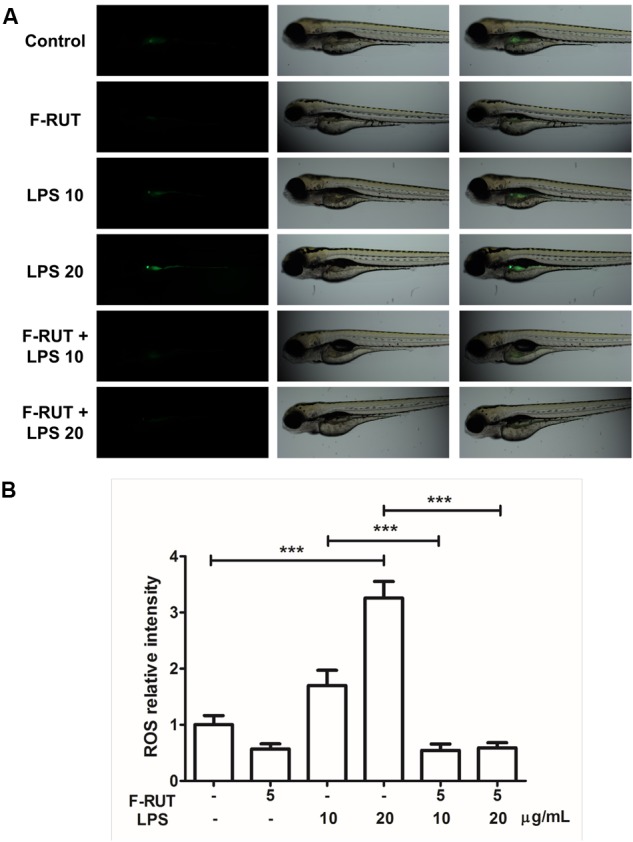
Fluoro-2-methoxyrutaecarpine (F-RUT) suppressed lipopolysaccharide (LPS)-induced reactive oxygen species (ROS) in zebrafish. **(A)** ROS generation in zebrafish was evaluated after treatment with LPS for 24 h at 10 and 20 μg/mL, and ROS were suppressed by F-RUT at 5 μg/mL. **(B)** The fluorescence intensity was quantified, and the values expressed as mean ± SD; ^∗∗∗^*p* < 0.001 (*n* = 8 or 9 per group).

Zebrafish has been widely used in developmental biology, drug discovery, oxidative stress response, and toxicology (Craig et al., 2007; Stewart et al., 2014). In this study, the anti-inflammatory activity of F-RUT was also evident in zebrafish (Figure 5A). LPS significantly induced ROS in zebrafish at 10 and 20 ng/mL (^∗∗∗^*p* < 0.001, compared to the control group). ROS level can be detected in zebrafish as the intensity of the green color increases with LPS concentration. Pretreatment with F-RUT at 5 ng/mL suppressed LPS-induced ROS to a similar level as the control group (^∗∗∗^*p* < 0.001, compared to the LPS group) (Figure 5B). Taken together, these data further confirms the anti-inflammatory effect of F-RUT both *in vitro* and *in vivo*.

### Fluoro-2-Methoxyrutaecarpine (F-RUT) Ameliorates Lung Inflammation and Collagen Formation in KLF10-KO Mice

**FIGURE 6:**
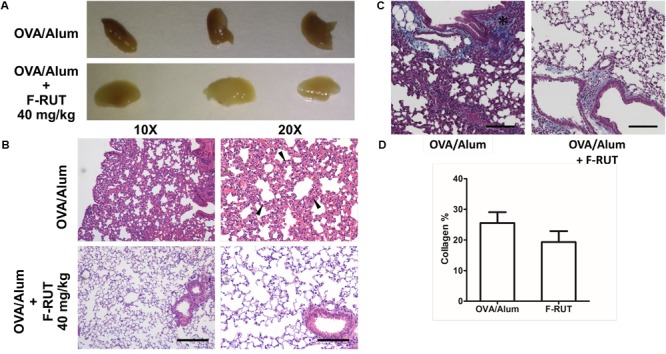
Fluoro-2-methoxyrutaecarpine (F-RUT) ameliorated inflammation in lungs of KLF-10-knockout (KO) mice. **(A,B)** KLF-10-KO mice were exposed to ovalbumin (OVA)/alum after treatment with 40 mg/kg F-RUT or PBS. Bar = 200 μm for the left panel and 100 μm for the right panel. **(C)** Masson’s trichrome stain was used to examine collagen formation. Suppression of the level of collagen after F-RUT treatment at 40 mg/kg was shown. **(D)** The intensity of collagen was normalized with the total area surrounding the bronchi in six random regions from each sample, and the average percentage was quantified. Bar = 100 μm (n = 3 per group).

*KLF10* gene is regulated by the transforming growth factor (TGF)-β/Smad. Deletion of KLF-10 in mice is associated with significant inflammation in the lungs when challenged with OVA/alum for 44 days (Huang et al., 2016). KLF10-KO mice also exhibits peripheral pro-inflammatory cytokine accumulation and displays enhanced CD4^+^ CD25 T cell activity, which promotes inflammation (Subramaniam et al., 2010). In this study, KLF-10-KO mice were employed to explore the anti-inflammatory effect of F-RUT. After OVA/alum challenge for 44 days, the lungs of treated mice showed inflammatory lesions; however, lung tissue of the F-RUT-treated group retained a normal morphological appearance. The difference in coloration, owing to the amount of inflammatory lesions, also indicated the anti-inflammatory effect of F-RUT (Figure 6A). With HE staining, lungs of challenged mice predominantly exhibited inflammation, as evidenced by the increased thickness of the alveolar wall (Figure 6B) and the increased number of infiltrating neutrophils (Figure 6B, black arrow) after challenging with OVA/alum for 44 days (Figure 6B). Alternate-day oral administration of 40 mg/kg F-RUT alleviated OVA/alum-induced lung inflammation and resulted in a normal alveolar wall structure (Figure 6B), indicating the protective effect of F-RUT. Collagen deposits were revealed by Masson’s trichrome staining (Figure 6C, blue-colored stain). The OVA/Alum group showed a darker and wider blue colored region, indicating higher collagen deposition activity (Figure 6C, left panel, asterisk). The addition of F-RUT, on the other hand, resulted in a reduced intensity of the blue color (Figure 6C, right panel). Moreover, we estimated the area of the blue-colored region, from which, the F-RUT-treated group revealed notable reduction compared to the OVA/alum challenged group (25% in the OVA/alum group and 18% in the F-RUT group) (Figure 6D).

### Fluoro-2-Methoxyrutaecarpine (F-RUT) Improves Inflammation-Stimulated Respiratory Interface in BALB/c Mice

**FIGURE 7:**
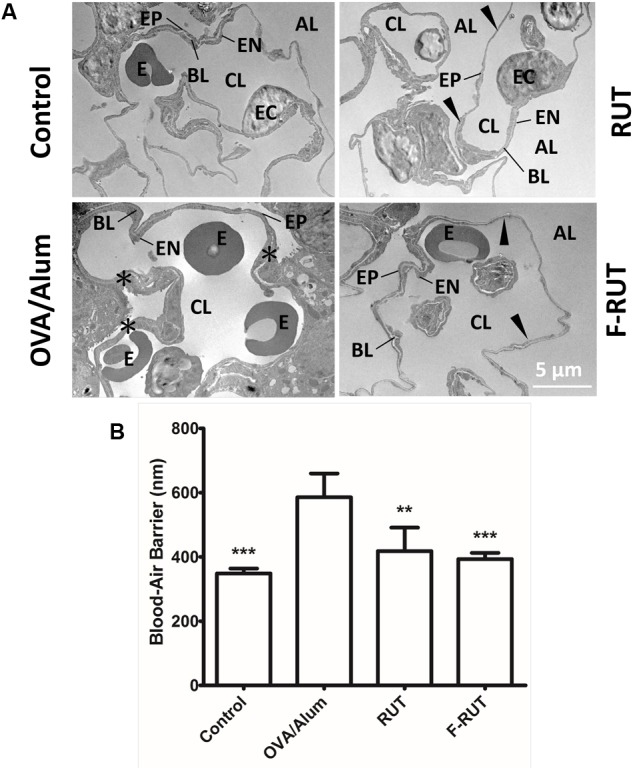
Fluoro-2-methoxyrutaecarpine (F-RUT) ameliorated the inflammation-stimulated respiratory interface in mice. **(A)** TEM image of the blood-air barrier in the lungs of BALB/c mice after being challenged with ovalbumin (OVA)/alum and an oral administration of 40 mg/kg rutaecarpine (RUT) or F-RUT. **(B)** The average thickness of the blood-air barrier, which is composed of the alveolar epithelium (EP), capillary endothelium (EN), and basal lamina (BL), was measured, and the results presented as mean ± SD; ^∗∗^*p* < 0.01, ^∗∗∗^*p* < 0.001 compared to the OVA/alum group. [E, erythrocyte; EC, endothelial cell (nucleus); CL, capillary lumen; AL, alveolar lumen; black arrow, blood-air barrier; asterisk, enlargement blood-air barrier; *n* = 4 or 5 per group].

The efficiency of the blood-air barrier (alveolar-capillary membrane), the gas-exchanging region of the lungs, was measured to determine lung function (Muhlfeld et al., 2007). After BALB/c mice were challenged with OVA/alum and orally administered RUT or F-RUT, lung tissues were harvested and the thickness of the blood-air barrier measured with TEM. The blood-air barrier is composed of the alveolar EP, EN, and BL (Figure 7A). Upon OVA/alum challenge, this barrier significantly thickened (Figure 7A, asterisk), with the addition of RUT and F-RUT reducing this thickness (Figure 7A, right upper and lower panel, respectively, black arrow). To better demonstrate the effect of F-RUT, the average thickness of the blood-air barrier was measured, and a significant increase in the OVA/alum-challenged group (238 nm in increased thickness compared to the control group) observed; this was reversed by treatment with RUT or F-RUT (at 70 and 45 nm in increased thickness and 70.6 and 81.1% reductions, respectively). More importantly, F-RUT showed a stronger protective effect than RUT, as F-RUT-treated mice displayed an additional 10.5% reduction in the blood-air-barrier thickness (Figure 7B). In essence, these data demonstrate a significantly stronger function of the reduction of lung inflammation by F-RUT, further proving that F-RUT can act as an anti-inflammatory drug.

## Discussion

In 2015, lower-respiratory infections and chronic obstructive pulmonary disease (COPD) were ranked third and fourth among the top 10 leading causes of death globally. Causes of pulmonary disease such as asthma and COPD are complex and may involve local inflammation. Side effects such as gastrointestinal disturbances, nausea, and vomiting, remains as major concerns from medical treatment. Pulmonary diseases are becoming a notable threat to public health, especially as air pollution remains a serious issue worldwide. Owing to this, the anti-inflammatory activity of RUT was previously explored (Chiou et al., 1992, 1997). F-RUT, the newly synthesized derivative of RUT, was previously shown to possess anti-inflammatory activity and exhibit lower cytotoxicity, spurring its pharmaceutical development (Lee et al., 2017). The present study explored the additional effects of F-RUT on respiratory function as well as morphological changes using a mice model. During inflammation, recruitment of immune cells and interactions with epithelial cells usually lead to enhanced adaptive immunity (Kato and Schleimer, 2007). However, excessive or persistent inflammation can lead to disease pathogenesis. For instance, TGF-β1 and TNF-α signaling are critical for collagen accumulation and fibrotic diseases (Saini et al., 2016). In addition, these pro-inflammatory cytokines contribute to myofibroblast transformation and migration (Wynn, 2011). KLF10 was also reported to suppress TGF-β-induced EMT (Mishra et al., 2017) and promote inflammation; KLF10 was also predominantly found in the lungs after challenge with OVA/alum (Subramaniam et al., 2010; Huang et al., 2016). KLF10-KO mice are therefore a suitable animal model to characterize *in vivo* anti-inflammatory activity. As levels of airway collagen were also found to correlate with lung function (Setlakwe et al., 2014), collagen formation around bronchi in OVA/alum-stimulated KLF-10-KO mice was observed and measured. Although there was no significant statistical change, a suppressive effect of F-RUT on collagen formation was observed (Figure 6B, coverage of collagen was 7% lower in the F-RUT group compared to the OVA/alum group). Furthermore, the blood-air-barrier thickness in mice was reduced by F-RUT after mice were challenged with OVA/alum. Taken together, these data demonstrated the capability of F-RUT to prevent constitutive inflammatory damage to the lungs, and thus, may be able to prevent fibrosis by reducing excessive collagen deposition.

Fluorine is widely used in drugs and bio-macromolecules for increased therapeutic efficacy (Muller et al., 2007; Purser et al., 2008). Potential applications of fluorine were also found in agrochemical, pharmaceutical, and material development (Yuan et al., 2017). Several fluorinated RUT compounds have been synthesized, but only F-RUT exhibited good anti-inflammatory activity (Lee et al., 2017). Results from a molecular docking simulation also proposed a mechanism used by F-RUT to exhibit its COX-2-inhibitory activity, and provided a fluorination position of molecular reference for pharmacokinetic purposes. A fluorine atom has a small steric size (42 pm); therefore, target proteins or enzymes may still be able to recognize fluorine-substituted molecules. In this study, a hydrogen atom at the 10C of F-RUT was substituted with fluorine. Based on the high electronegativity of fluorine, this reaction may provide an electron acceptor and reduce the strength of the electrophilic reaction in the indole structure by stabilizing the π electron of F-RUT. Therefore, F-RUT was synthesized as a stable compound that cannot be easily degraded and metabolized as RUT. This modification may also reduce intracellular reactivity to cell-maintenance substances and further prevent the occurrence of undesired oxidative metabolic processes (Van Heek et al., 1997; Liang et al., 2013). It may also be considered a beneficial pharmaceutical characteristic of F-RUT for drug preservation, sustenance in cells, and prolonged effective duration. Furthermore, a fluorine-modified compound could improve hydrophobicity and provide a binding force such as a hydrophobic pocket, to target proteins as was demonstrated in our molecular-simulation experiment. Overall, our potent fluorine-containing RUT displayed superior inhibitory and anti-inflammatory effects on COX-2. Such modification allowed F-RUT to exhibit both enhanced molecule reactivity and biological activity to provide a new strategy for fluorine-modified design in pharmaceutical production.

Aspirin, an NSAID, is widely used as a clinical anti-inflammatory drug and is also prescribed to prevent heart disease and stroke (Lewis et al., 1983). Aspirin, however, non-selectively and irreversibly inhibits both COX-1 and COX-2 via the acetylation of serine residues (Vane and Botting, 2003). COX-1 participates in the regulation of gastric mucosal blood flow, whereas COX-2 regulates the adhesion of white blood cells and endothelial cells. Therefore, simultaneous blockage of COX-1 and COX-2 usually leads to extreme side-effects in the gastrointestinal tract (Fiorucci et al., 2003). Therefore, a new type of NSAID and selective COX-2 inhibitor would be preferred to reduce these side effects (Warner and Mitchell, 2002). However, a selective COX-2 inhibitor was also reported to increase the risk of heart disease, which led to the recall of two commercial drugs, Vioxx and Bextra (Bahmani et al., 2017). The possible mechanism may have involved a relatively lower PGI_2_ than thromboxanes following COX-2 inhibition, which reduced the anticoagulation ability. For many years, the traditional Chinese herbal medicine, *E. rutaecarpa*, has been used to treat gastrointestinal disorders and headache (Jia and Hu, 2010). F-RUT is expected to retain this benefit as well as exhibit anti-inflammatory activity in gastrointestinal disorders. F-RUT was also found to have a potential application in the improvement of cardiac function and vasodilation by activating endothelial transient receptor potential vanilloid-type 1 (Lee et al., 2017). In addition, a weaker COX-2 inhibition effect of F-RUT than celebrex may not drive the decline in protective vascular PGI_2_ to a harmful level. Taken together, these data demonstrate that F-RUT can be a good alternative COX-2-selective inhibitor owing to fewer side effects than other inhibitors.

Cyclooxygenase (COX)-2-selective inhibitor is an effective anti-inflammatory drug; however, it may increase the risk of side effects. Fluorine-containing organic compounds have been applied in a wide range of pharmaceutical products. Fluorination of RUT is expected to diminish π electron delocalization and decrease the charge density at the ring center, thereby enhancing metabolic stability by hampering undesired oxidative metabolic pathways. F-RUT was demonstrated as a COX-2-selective inhibitor and in this study, the anti-inflammatory activity of F-RUT was displayed *in vitro* and *in vivo*. Furthermore, the ability of F-RUT to protect the lungs was demonstrated following inflammatory challenge in KLF10-KO mice. The enhanced bioactivity and lower cytotoxicity of F-RUT are advantageous to conquer the undesirable side effects, for instance, in gastrointestinal or heart diseases. Taken together, F-RUT, a derivative of a natural compound, is a better option for use in pharmaceutical applications as a new class of COX-2 inhibitors than conventionally used compounds.

## Author Contributions

ChiL, CY, and JL performed the experiments and analyzed the data. SC and YL performed the experiments. ShH contributed to reference search and coordination. ChiL contributed to experiment design and manuscript preparation. StH contributed to strategic design of synthetic drug. VC contributed to animal model design. ChuL conducted research.

## Conflict of Interest Statement

The authors declare that the research was conducted in the absence of any commercial or financial relationships that could be construed as a potential conflict of interest.

## Footnotes

1 https://www.rcsb.org
