# Supplementary figures and images for: Fluorine-Modified Rutaecarpine Exerts Cyclooxygenase-2 Inhibition and Anti-inflammatory Effects in Lungs
Source: Front Pharmacol. 2019 Feb 7;10:91. doi: 10.3389/fphar.2019.00091 (PMC6374341; doi:10.3389/fphar.2019.00091)

**Table 1. The inhibitory activity of F-RUT and RUT on COXs.**


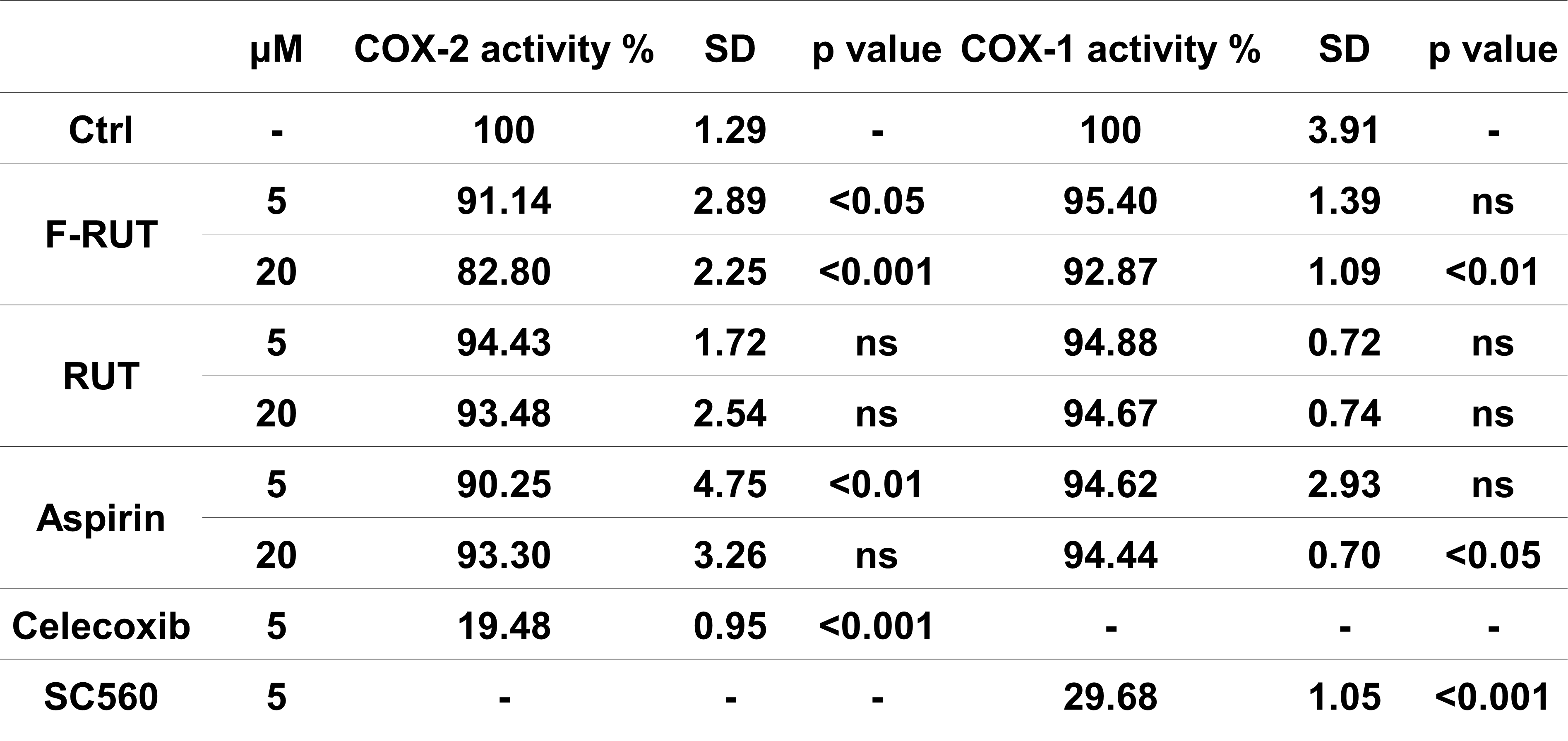

Supplement: Supplementary file 1 [file Table_1.DOCX]

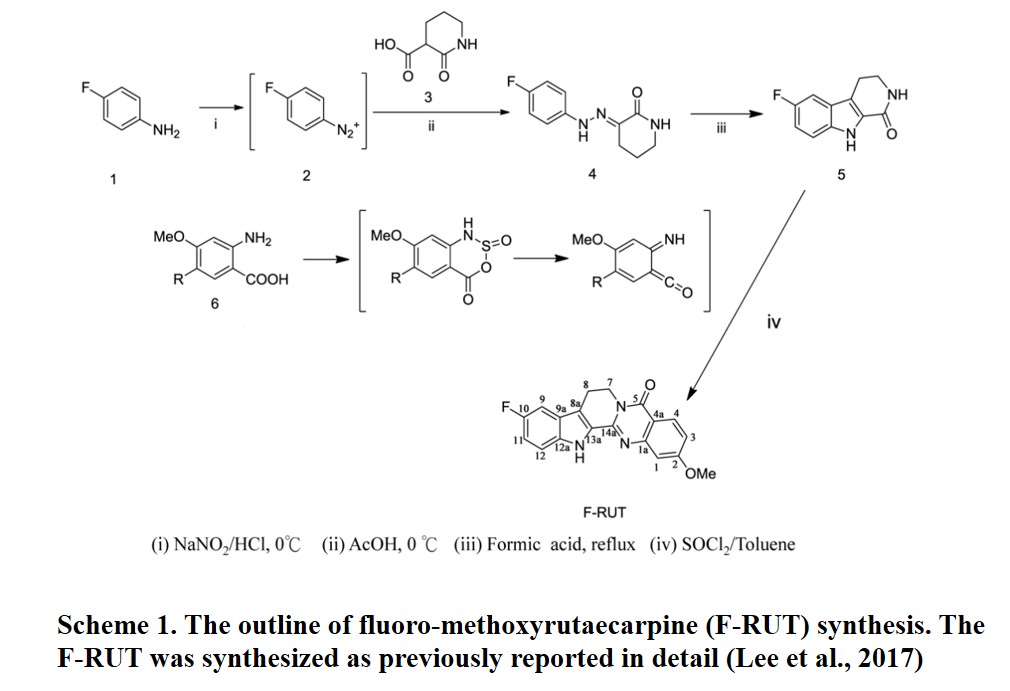

Supplement: Supplementary file 2 [file Image_1.JPEG]
